# Effects of Food Depictions in Entertainment Media on Children’s Unhealthy Food Preferences: Content Analysis Linked With Panel Data

**DOI:** 10.2196/51429

**Published:** 2024-05-22

**Authors:** Jörg Matthes, Alice Binder, Brigitte Naderer, Michaela Forrai, Ines Spielvogel, Helena Knupfer, Melanie Saumer

**Affiliations:** 1 Department of Communication, University of Vienna, Vienna, Austria; 2 Department for Social and Preventive Medicine, Medical University Vienna, Vienna, Austria

**Keywords:** children, health, unhealthy food preferences, food depictions, centrality, coviewing, longitudinal linkage study, child, food, eating, diet, dietary, preference, preferences, nutrition, nutritional, diet, media, entertainment, panel, foods, pediatric, pediatrics, food preference, food preferences

## Abstract

**Background:**

Entertainment media content is often mentioned as one of the roots of children’s unhealthy food consumption. This might be due to the high quantity of unhealthy foods presented in children’s media environments. However, less is known about the role of the centrality of food placement, that is, whether foods are interacted with, consumed, verbally mentioned, or appear unobtrusively. We also lack longitudinal research measuring both children’s unhealthy and healthy food consumption behaviors as outcomes.

**Objective:**

The aim is to connect content analytical data based on children’s actual media diet with panel data in order to explain children’s food preferences. Moreover, this study not only focuses on the amount of healthy and unhealthy foods children are exposed to, but also on how these foods are presented (ie, centrally or not). Furthermore, we looked at the question of how parental coviewing can diminish (or enhance) the effects of unhealthy (or healthy) food depictions, and we measured healthy and unhealthy consumption as dependent variables.

**Methods:**

We conducted a 2-wave panel study with children and one of their parents (of 2250 parents contacted, 829 responded, for a response rate of 36.84%; 648 valid cases, ie, parent-child pairs, were used for analysis), with 6 months between the 2 panel waves. We linked the 2-wave panel data for the children and their parents to content analytical data for movies (n=113) and TV series (n=134; 3 randomly chosen episodes per TV series were used) that children were exposed to over the course of 6 months.

**Results:**

There was no significant relationship between exposure to unhealthy food presentation and unhealthy (b=0.008; *P*=.07) or healthy (b=−0.003; *P*=.57) food consumption over time. Also, healthy food presentation was unrelated to unhealthy (b=0.009; *P*=.18) or healthy (b=0.000; *P*=.99) food consumption over time. However, there was a significant, positive interaction between unhealthy food presentation and presentation centrality on unhealthy food consumption (b=0.000; *P*=.03), suggesting that the effects of unhealthy food presentation rise with increasing levels of centrality. There was no interaction between unhealthy food presentation and presentation centrality on the consumption of healthy foods (b=0.000; *P*=.10). Also, exposure to healthy food presentation interacted with centrality (b=−0.001; *P*=.003). That is, when a healthy product was presented at maximum centrality, it led to less unhealthy food consumption in children. Coviewing did not interact with exposure to unhealthy foods when explaining unhealthy (b=0.003; *P*=.08) or healthy (b=−0.001; *P*=.70) food consumption.

**Conclusions:**

We conclude that simply presenting more healthy foods is not sufficient to combat children’s unhealthy food preferences. Further regulations may be necessary with respect to representations of unhealthy foods in children’s media.

## Introduction

### Background

There is a great debate among parents, teachers, politicians, and marketers as to how the media contributes to the development of childhood obesity [1]. Recently, meta-analytic data [2-4] and literature reviews [1,5] summarizing a large corpus of empirical studies have suggested that children’s confrontation with media content, particularly persuasive content—such as traditional TV commercials [6,7], product placements in movies [8], brand presentations in YouTube videos [9], or online advertisements [10,11]—impacts their food preferences and eating behaviors, particularly regarding the consumption of foods high in fat, salt, or sugar.

Media content creates a very narrow food environment for children. Most of the content children are exposed to presents foods high in fat, salt, or sugar [12-17]. This kind of content is not only shown most often, the characters in children’s movies also consume these items most frequently and evaluate them predominantly very positively [15]. The consumption of highly processed products that are high in fat, salt, or sugar but low in nutritional value (ie, minerals or vitamins) is contributing to the development of overweight and obesity and therefore can be categorized as unhealthy. The overrepresentation of these foods in audiovisual media content is indeed worrying [18].

Drawing on the notion of cue reactivity, this paper describes a nonexperimental study linking content analytical data with panel survey data on the food preferences of children. We first review the literature on media effects on children’s food preferences and then explain the goals, methods, and findings of the study.

### Prior Work

Regarding children’s media environment, it has been argued that products integrated within editorial content, interrupting the content, can be more influential on product choices than classical advertisements [19]. This is especially true for children because they are still developing their cognitive abilities and might therefore not be aware of attempts to persuade them that are integrated with the entertainment content [20]. A study on the Reactivity of Embedded Food Cues in Advertising Model (REFCAM) [21] found that when such presentations were integrated within the editorial content, it first led to a kind of cue reactivity. This cue reactivity revealed itself with an increased heart rate [22] or a higher likelihood of thinking about the presented product [23]. In the next step, this cue reactivity influenced children’s eating habits [21]. The authors described this pathway as being influenced by individual susceptibility factors, such as children’s BMI or age, and also by “the level of integration” of a product [21]. Thus, how a product is presented might influence children’s reactions.

Although scholars have theorized that the “food-related media diet” is also represented in children’s actual diet, this assumption has never been formally tested to date. The available evidence comes from experimental and survey research focusing on unhealthy [24-28] and healthy foods [28-30]. Whether healthy food presentations have the power to influence healthy food choices is not entirely clear from current empirical evidence [19,31].

It might not be sufficient to only consider what food is presented in media content targeted at children; it will also be necessary to consider how the presented food is shown [32]. Since drawing attention to the food is crucial for arousing appetite, reinforcing appreciation for that food and thus triggering eating behaviors [21,33] is more likely when the depicted food plays a central role in the media content. Therefore, presentation centrality, which is commonly connected to interaction with a product (ie, whether one of the characters on screen handles a product, consumes it, or verbally mentions it) [34], is an important factor that needs to be considered. A study by Charry [30] showed that audiovisual presentations of fruit led to higher intentions of choosing fruit compared to a presentation that was only visual. Similarly, findings by Naderer and colleagues [35] suggest that a character handling or consuming the food elicits higher food consumption for the presented snack compared to the food being only visually presented.

Furthermore, social factors can play a crucial role in influencing obesity in children [36]. Parents heavily shape their children’s food environment and thus play a significant role in both establishing children’s food preferences and gatekeeping the media food environment of their children [37]. The theory of parental mediation states that parents’ behaviors can influence how children access, receive, process, and react to media content and to what extent children adopt behaviors presented in media [38]. Parental mediation encompasses a variety of distinct social behaviors [39] that can be important in the prevention of negative media effects in children [40]. One behavior is called social coviewing, which is the joint viewing of media content by parents and children without necessarily talking about the content [39,41].

Experimental data measuring food choices and eating behaviors shortly after exposure to persuasive content suggest that media depictions do significantly shape unhealthy eating behaviors among children [3,4]. However, as valuable as experimental studies are, they do not allow conclusions about long-term effects, and they typically test specific food depictions, not the food-related media diet as a whole. Cross-sectional data examining the correlation of media consumption and children’s BMI indicate that children with factors such as extensive TV viewing also are more prone to be overweight or obese [42]. Some first longitudinal examinations also speak to this relationship [43,44]. However, these studies do not take into account the actual content that children are exposed to.

### Goal of This Study

With an extensive and externally valid design, this study aimed to link content analytical data on children’s actual audiovisual media content—that is, food depictions in movies and TV series they were exposed to—with their food preferences, measured with survey data. This study used panel data from children and their parents over 6 months. The content analytical data represent the appearance and centrality of unhealthy foods, but also—as another urgent area for research—those of healthy foods. The survey data included parental coviewing measures that may diminish (or enhance) the effects of unhealthy (or healthy) food depictions. As dependent variables, unhealthy, but also healthy, food consumption of children was integrated within the model.

## Methods

### Ethical Considerations

The study was approved by the ethics committee of the University of Vienna (00343) and the principals of the respective schools. Prior to each wave, the children were asked to take home an information sheet, a consent form, and a survey for their parents. All children who returned a consent form that was signed by one of their parents at the first measurement point (T1) were then asked for their oral consent; if they agreed, we interviewed them individually and measured their weight and their height. Data were anonymized.

### Procedure

#### Overview

This longitudinal linkage study combined survey data from parents and children with content analytical data. A 2-wave panel study was conducted with children and 1 of their parents (n=648) with 6 months in between the 2 panel waves. The study combined this approach with a content analysis of movies (n=113) and TV series (n=134; 3 randomly chosen episodes per TV series) that the children or their parents indicated they had been watching during the last 6 months at the second measurement point (T2).

#### Panel Survey

The data for this study originated from a larger project for which a 2-wave panel survey was conducted among a convenience sample of children and parents who were recruited via primary schools in Austria.

Parents were told that the purpose of the study was academic and that participation was voluntary. We ensured that both the children and their parents were aware that they could withdraw their consent or choose not to answer at any point in time. All parent surveys that were returned were then matched with the data provided by the children. This procedure was followed for both waves. The data for the first wave were collected from March to May 2019; data for the second wave were collected 6 months later.

Initially, the required documents were handed out to approximately 2250 children in 12 primary schools (n=6, 50% of the schools were in urban areas and n=6, 50% in rural areas). Overall, the response rate was 36.84%; thus, initially, 829 children returned the signed informed consent form. Due to some cases of illness and other issues, 795 children were interviewed at T1; 734 of the children at T1 participated again at T2. Comparing the children who remained with those who dropped out revealed no significant difference with respect to gender (*P*=.96), age (*P*=.66), or BMI (*P*=.29)

Moreover, 778 of the children’s parents again returned a questionnaire at T2. For the analysis, our primary interest was the data from the children who participated in both waves (n=734). However, the parents’ answers were also important to obtain a good picture of what audiovisual content children watched during the 6 months between the 2 measurement points. Overall, 559 parents at T2 filled out the questions concerning their children’s consumption of audiovisual content. To construct a meaningful linkage, only cases with at least 3 named movies or TV series over the past 6 months according to the data provided by children or by their parents were included. Following this procedure, the final analyses were based on the data of 648 children aged between 5 and 11 years (T1: mean age 7.78, SD 0.50 years [n=15 missing]; n=313, 48.3% female [n=17 missing]; T2: mean age 8.26, SD 1.24 years; n=313, 48.4% female).

#### Content Analysis

To determine the sample for the content analysis, the movies and TV series that were named by children and parents in the open-ended questions about past media consumption and the parents’ selections from a list in the parental survey at T2 were combined. All movies and TV series that were watched by at least 5 children were considered. Furthermore, audiovisual content named by a child or a parent was only coded if the child had watched at least 3 different movies or TV series. However, to avoid excessive dropout, movies and TV series were included if they were mentioned by fewer than 5 children if at least 1 of them had not mentioned at least 3 other movies or TV series.

The following criteria were applied to determine whether the movies and TV series were relevant for this study: First, all movies and TV series were excluded that were inappropriate for children according to their age rating (we included age ratings up to 12 years). Second, all media content was excluded that did not have a clear storyline; hence, game shows, educational TV series, and cooking shows were removed. Third, all media content was excluded that was not available in German. Finally, movies and TV series were excluded that were not available on streaming platforms or on DVD/BluRay. If multiple movies from the same movie series had been named, a randomizer selected one, again taking into account their availability on streaming platforms or via the Vienna Public Libraries; also, a randomizer selected 3 episodes from each TV series following the same procedure. The final sample consisted of 113 movies and 3 episodes each from 134 named TV series.

### Measures

#### Exposure to Unhealthy and Healthy Products

To determine the independent variable, that is, the children’s audiovisual content exposure (ie, movies or TV series) within the 6 months between the 2 waves, the children and their parents were asked which movies and TV series they had seen during this period. In addition to this open-ended question, the parental survey also contained a list of (at the time) popular audiovisual media content for children, as well as content specifically directed at children that was currently available on Netflix (Netflix, Inc) or Amazon Prime Video (Amazon.com, Inc, from which parents were able to choose.

Then, all food and beverage placements within each scene of the 113 movies and 3 episodes from each of 134 TV series were coded; scenes were defined as 5-minute segments. Five coders were extensively trained and, after completing a total of 2 rounds of reliability testing involving a total of 450 scenes, were deemed reliable (healthfulness of the product: Krippendorff α=0.79; food presentation centrality: Krippendorff α=0.85).

Each food placement (n=12,358) was either deemed healthy, unhealthy, or mixed. This differentiation was based on the recommendations of the World Health Organization [45] and former studies as follows: (1) unprocessed products with a high nutritional value were categorized as healthy, including water, unsweetened tea and juice, and fruit and vegetables (n=2953, 23.9% placements); (2) highly processed products high in fat, salt, and sugar were categorized as unhealthy, including sweets, french fries, and soft drinks (n=4926, 39.7%); and (3) products that included healthy as well as unhealthy ingredients were categorized as mixed products, including combined meals (n=4479, 36.2%). However, this study focused on the exposure effects of healthy or unhealthy products.

The total number of healthy and unhealthy placements within each watched movie or TV series (in the latter case, we added up the data from the 3 episodes we coded) per child was calculated; each child saw a mean 20.87 (SD 9.36) unhealthy products and a mean 11.23 (SD 4.43) healthy products.

Additionally, food placement centrality, that is, whether the placement was a focal point (if one of the characters on screen interacted with the product, consumed it, or verbally mentioned it, it was scored a 1; if it was not a focal point, it was scored a 0) was coded. Overall, 7738 (62.4%) product references appeared as focal points. The total number of focal-point placements within each watched movie and TV series per child was calculated (mean 36.99, SD 13.25 placements).

#### Coviewing

Based on on the work of Valkenburg and colleagues [41], parental coviewing was measured with 4 items on a 7-point Likert scale at T1 (1=never to 7=very often [in response to the question “How often do you watch a movie/TV series together with your child because you both like it?”]; at T1: Cronbach α=0.82; mean score 4.51, SD 1.47) in the parental survey.

#### Food Preferences

The dependent variables were measured in the panel survey of the children. We assessed how often the children consumed unhealthy food with 4 items (“How often do you eat [drink] sweets/salty snacks/soft drinks?”; 1=never to 4=very often; at T1: Cronbach α=0.65; mean score 2.20, SD 0.60; at T2: Cronbach α=0.68; mean score 2.19, SD 0.57). Furthermore, the children’s healthy food consumption was assessed with 3 items (“How often do you eat [drink] fruit/vegetables/water?”; 1=never to 4=very often; at T1: Cronbach α=0.54; mean score 3.36, SD 0.56; at T2: Cronbach α=0.61; mean score 3.41, SD 0.55). In an additional analysis, water was excluded from the index, which did not affect the findings reported in the Results section.

#### Control Variables

The children’s BMI at T1 was included as a control variable. Therefore, the children’s weight and height were measured. For the analyses, their zBMI (SD score of BMI [46]) was calculated to adjust their BMI to their age and their gender (T1: n=628; zBMI: mean 0.09, SD 1.12; n=90, 14.3% overweight; n=33, 5.6% obese; n=20 missing). Furthermore, the children’s overall audiovisual media exposure was included as a control variable. Children’s media consumption was assessed at T2 using 2 items that respectively focused on movies (“How many movies are you allowed to watch on TV or on the internet at home in one week?”; 1=none, 2=one during the weekend, 3=one per day, 4=as many as I want) and TV series (“How many series are you allowed to watch on TV or on the internet at home in one week?”; 1=none, 2=one episode; 3=several episodes; 4=as many as I want). The items formed a reliable index (Cronbach α=0.63; mean 2.58, SD 0.65). Furthermore, we controlled for the children’s age (mean age 7.78, SD 0.50 years; n=15 missing) and gender (n=313, 48.3% female; n=17 missing).

Table 1 provides an overview of variables used in the models.

**Table 1.** Description of measured variables.

Variables	Values, mean (SD)
Unhealthy food consumption (score; wave 1)	2.20 (0.60)
Unhealthy food consumption (score; wave 2)	2.19 (0.57)
Healthy food consumption (score; wave 1)	3.36 (0.56)
Healthy food consumption (score; wave 2)	3.41 (0.55)
Age (years; wave 1)	7.78 (0.50)
BMI (score; wave 1)	0.09 (1.12)
Media consumption (score; wave 2)	2.58 (0.65)
Unhealthy food presentations (n; wave 2)	20.87 (9.36)
Healthy food presentations (n; wave 2)	11.23 (4.43)
Focal-point placements (n; wave 2)	36.99 (13.25)
Coviewing (score; wave 1)	4.51 (1.47)

### Statistical Analysis

A moderated regression analysis was performed controlling for healthy or unhealthy food consumption as autoregressive paths. All predictors were entered simultaneously and terms were mean-centered prior to computing interaction terms.

## Results

The expectations were that exposure to unhealthy food products would be (1) positively related with unhealthy food consumption and (2) negatively related with healthy food consumption. Neither of these expectations found support. There was no significant relationship between exposure to unhealthy food presentations and unhealthy (b=0.008; *P*=.07) or healthy (b=−0.003; *P*=.57) food consumption over time. Moreover, this study examined how healthy food presentations in children’s media would relate to unhealthy and healthy food consumption over time. As indicated in Table 2, there were no significant relationships (unhealthy food consumption: b=0.009; *P*=.18; healthy food consumption: b=0.000; *P*=.99).

**Figure 1.**
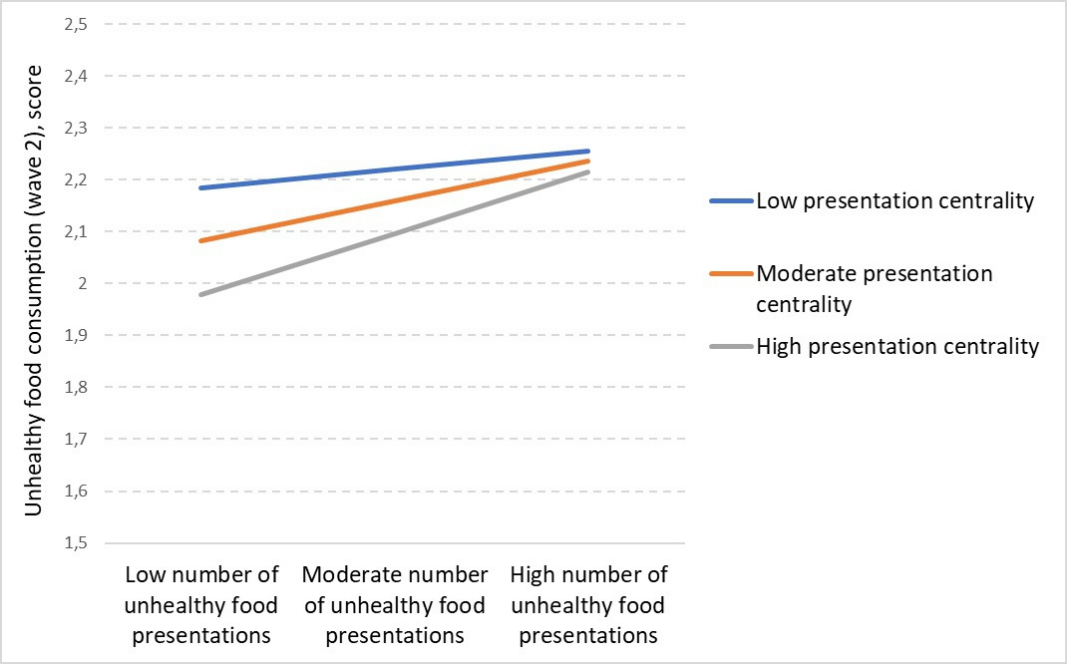
Effects of unhealthy food presentations on unhealthy food consumption by presentation centrality.

We found that exposure to unhealthy food presentations and presentation centrality had a significant positive interaction effect with unhealthy food consumption (b=0.000; *P*=.03). The positive sign of the interaction suggests that the effects of unhealthy food presentation rose with increasing levels of centrality. The probing of the interaction [47] is shown in Figure 1: starting from a level of presentation centrality of 1.65, the relationship is significantly positive (b=0.088; *P*=.05) and rises to an effect of b=.034 (*P*=.03). However, unhealthy food presentation and presentation centrality had no interaction with the consumption of healthy foods (b=0.000; *P*=.10).

**Figure 2.**
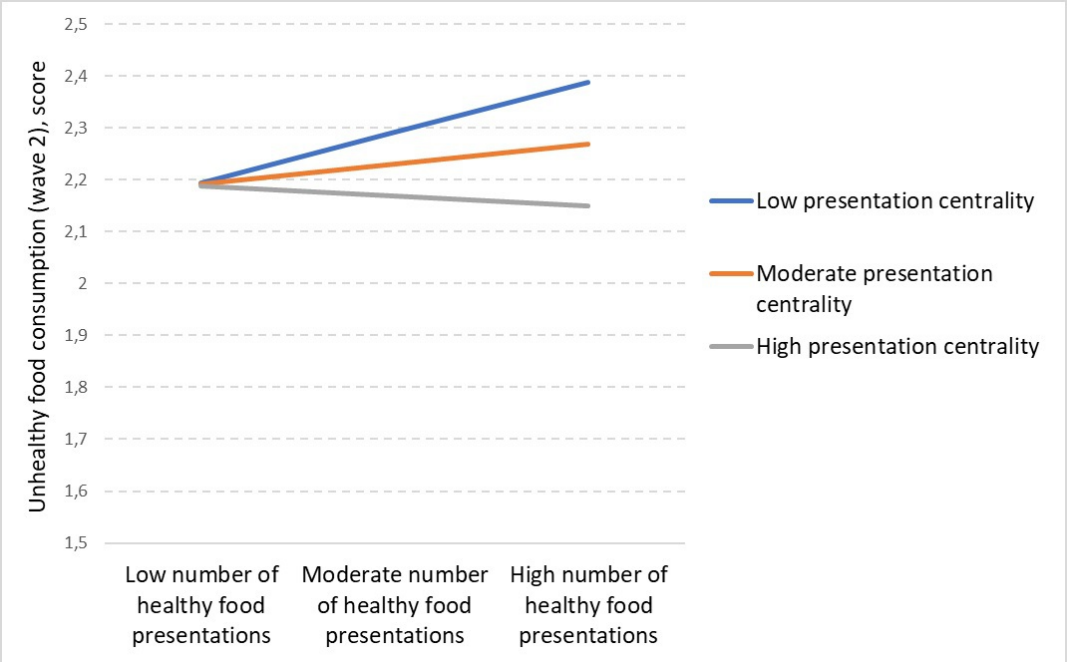
Effects of healthy food presentations on unhealthy food consumption by presentation centrality.

When it comes to healthy food presentations, there was also a significant interaction with centrality (b=−0.001; *P*=.003). As can be seen in Figure 2, only for low food presentation centrality, there was an effect of exposure to healthy foods on unhealthy food consumption. Probing of this interaction revealed that for a mean-centered centrality lower than −5.196, the effect of healthy food presentations was significantly positive (*P*<.05) and was largest for the lowest centrality (b=0.40; *P*=.003). For values of centrality higher than 31.434, the effect turned significantly negative (*P*<.05) and was largest for the highest centrality (b=−0.07; *P*=.009). This means that when a healthy product was presented at maximum centrality, it led to less unhealthy food consumption in children. In contrast to expectations, healthy food presentation and presentation centrality had no interaction with healthy food consumption (b=−0.000; *P*=.22). Presentation centrality had no significant relationship with unhealthy food consumption (b=−0.005; *P*=.21) or healthy food consumption (b=0.001; *P*=.71).

**Table 2.** Unstandardized coefficients for predicting unhealthy food consumption (n=591; *R²*=0.299).

Predictors	Unhealthy food consumption (wave 2)	
	b (SE)	*P* value^a^
Constant	1.608 (0.175)	<.001
Unhealthy food consumption (wave 1; autoregressive)	0.384 (0.037)	*<*.*001*
Gender	0.087 (0.041)	.*03*
Age (wave 1)	−0.050 (0.017)	.*004*
BMI (wave 1)	−0.014 (0.018)	.43
Media consumption (wave 2)	0.111 (0.034)	.*001*
Unhealthy food presentation (wave 2)	0.008 (0.005)	.07
Healthy food presentations (wave 2)	0.009 (0.007)	.18
Presentation centrality (wave 2)	−0.005 (0.004)	.21
Coviewing (wave 1)	0.044 (0.013)	.*001*
Unhealthy food presentations × presentation centrality	0.000 (0.000)	.*03*
Unhealthy food presentations × coviewing	0.003 (0.002)	.08
Healthy food presentations × presentation centrality	−0.001 (0.000)	.*003*
Healthy food presentations × coviewing	−0.002 (0.003)	.60

a Significant *P* values are italicized.

Coviewing did not interact with exposure to unhealthy products in audiovisual media when explaining unhealthy (b=0.003; *P*=.08) or healthy (b=−0.001; *P*=.70) food consumption behavior of children over time. It also did not interact with the presentation of healthy products with regard to unhealthy (b=−0.002; *P*=.60) or healthy (b=0.001; *P*=.74) food consumption. Surprisingly, coviewing was associated with higher unhealthy food consumption over time (b=0.044; *P*=.001), but it was unrelated to healthy food consumption (b=0.005; *P*=.69).

As for the controls, boys were more likely than girls to consume unhealthy foods, and age was positively correlated with unhealthy food consumption. Both gender and age were unrelated to healthy food consumption. Overall media consumption was positively related to the consumption of unhealthy foods and unrelated to healthy food consumption. Children’s zBMI had no relationship with either type of food consumption. Tables 2 and 3 show detailed findings from the controls.

**Table 3.** Unstandardized coefficients for predicting healthy food consumption (n=591; *R²*=.322).

Predictors	Healthy food consumption (wave 2)	
	b (SE)	*P* value^a^
Constant	1.431 (0.175)	<.001
Healthy food consumption (wave 1; autoregressive)	0.537 (0.035)	*<*.*001*
Gender	0.010 (0.040)	.80
Age (wave 1)	0.021 (0.017)	.21
BMI (wave 1)	−0.015 (0.017)	.40
Media consumption (wave 2)	−0.020 (0.032)	.53
Unhealthy food presentations (wave 2)	−0.003 (0.004)	.57
Healthy food presentations (wave 2)	0.000 (0.006)	.99
Presentation centrality (wave 2)	0.001 (0.004)	.71
Coviewing (wave 1)	0.005 (0.012)	.69
Unhealthy food presentations × presentation centrality	0.000 (0.000)	>.99
Unhealthy food presentations × coviewing	−0.001 (0.001)	.70
Healthy food presentations × presentation centrality	−0.000 (0.000)	.22
Healthy food presentations × coviewing	0.001 (0.003)	.74

a Significant *P* values are italicized.

## Discussion

### Principal Results and Comparison With Prior Work

Evidence is abundant for the effects of unhealthy food presentation in children’s media on children’s consumption behaviors [4,19,48]. The aim of this study was to revisit this evidence, not only with respect to the presentation of unhealthy foods, but also healthy ones. For the first time in the existing research, content analytical data from movies and TV series that children were exposed to over a time span of 6 months were combined with panel data from children and their parents. With this data linkage design, this study could test how effects evolved over time.

As the findings reveal, even though unhealthy food exposure was much higher overall, exposure to unhealthy and healthy foods alone was not related to children’s healthy and unhealthy consumption behaviors. The results show that the centrality with which the products were presented greatly mattered for the relationships. When foods were presented centrally, that is, when they were zoomed in on or when the characters interacted with them, consumed them, or verbally mentioned them, exposure to unhealthy food presentation was positively related to unhealthy food consumption. This effect can be explained by the fact that centrality eases the perception of unhealthy foods, thus increasing cue reactivity, leading to a “wanting” of that product [33]. That is, centrality plays a role in reminding children about their food preferences.

Interestingly, this relationship was different for the presentation of healthy foods. For healthy foods, centrally placed healthy food products were negatively related to unhealthy food consumption. One could argue that children are made aware of the importance of healthy foods for their diet and thus their preference for unhealthy foods decreases. Yet to create such an awareness, a central placement of the food is necessary. Of course, more empirical evidence is needed to corroborate that claim.

However, when healthy foods were placed noncentrally, they were positively related to children’s consumption of unhealthy food over time. This finding is in line with a prior study [19] suggesting that healthy food placement can promote unhealthy eating behaviors. Subtle presentation of healthy foods can activate children’s inherent preference for unhealthy foods, for instance, by serving as a cue for appetite [32]. When appetite is cued, then children automatically prefer unhealthy over healthy options. However, when the centrality of healthy foods rises, this automatic process may be impeded; children may be reminded about the importance and necessity of healthy food and consume less unhealthy food over time. Again, the precise underlying mechanisms remain to be studied. Overall, even though unhealthy foods were presented more often, the amount of food exposure was not a key element influencing unhealthy food consumption. However, persuasive strategies (ie, centrality) in connection with unhealthy as well as healthy food presentation are more important in that regard.

Against expectations, foods presented in the media that children were exposed to did not show any relationship to healthy food consumption. Healthy food consumption could hardly be explained empirically. One explanation could be that children have an inherent preference for unhealthy foods [49,50]. As a consequence, unhealthy food consumption may be triggered more easily as compared to healthy food consumption. This is also in line with former research testing the effects of different persuasive strategies concerning healthy food consumption [23,51,52]. It seems that more than just a central placement is needed to positively impact children’s healthy eating habits [32].

Finally, coviewing by parents did not moderate the effects on healthy and unhealthy food consumption; however, it was positively related to unhealthy food consumption overall. This finding is arguably hard to explain, as theory would suggest the opposite. Perhaps coviewing exerts an indirect effect: when coviewing, parents, too, are exposed to unhealthy food presentations (which are clearly dominant in children’s media) [15], and they may also be affected by them. These effects on parents may then, in a second step, facilitate the unhealthy eating habits of their children. In such a scenario, potential effects on children could run via 2 paths: unhealthy food presentation could shape children’s eating behaviors directly (ie, by affecting them), and indirectly (ie, by affecting their parents, who then affect their children). This indirect mechanism is certainly speculative and cannot be properly tested in a panel survey; it would thus necessitate strict experimental designs.

### Limitations and Future Research

This study relied on self-reported data when assessing the specific movies and TV series that children were exposed to. As always, such self-reported data are prone to perceptual biases, as specific movies and TV series may be more likely to be mentioned for reasons that cannot be measured. However, considering that in this study both children and parents were asked about movies and TV series, and content viewed over 6 months would generally be possible to remember, we are confident that we obtained an account of the content the children were exposed to. Also, when it comes to the dependent variables, self-reported consumption behaviors, which are not the same as actual food choices, were assessed [53]. Related to that, this study can only make correlative conclusions regarding the relationship between mediated exposure to foods and children’s consumption behaviors.

The sample relied on the most frequently was movies and TV series. However, this study was unable to include smartphone content or determine the type of screen on which the content was consumed. For the age group of this study (between 5 and 11 years) and their typical content preferences, this seemed reasonable. In addition, the models controlled for overall media consumption to rule out effects of the frequency of exposure to other content not assessed in this study, but future research should take a 360 degree account of children’s media diet. This may be challenging for practical reasons, but integrating several types of content in one study is important. Relatedly, we did not predict reciprocal relations (ie, food presentation by consumption), because food presentation refers not only to exposure, but also to content, and the content of the movies cannot be predicted by consumption. However, future research should follow up on this.

This study focused on the centrality of presentation. As important as centrality is [33], it is only one presentation factor that may shape food preferences [32]. Also, this study did not explicitly distinguish between several types of centrality, such as interacting with a product, consuming it, or mentioning it. When it comes to coviewing, several dimensions should also be distinguished in future research, such as intentional versus passive coviewing. Moreover, the age range used in this study deserves further scrutiny. Children younger than 5 years are frequently exposed to media content containing food. These children are, arguably, particularly susceptible to the presentation of unhealthy and healthy food and thus deserve more attention. Finally, no data on the specific schools the children were attending were collected since these would not have been in line with the ethical standards regarding anonymization. Therefore, this study was not able to control for schools in the models.

### Conclusions

When discussing the roots of children’s unhealthy food consumption and childhood obesity, educators, journalists, and policy makers have been very quick to point their fingers at the media, particularly media that targets a young audience. At first sight, the evidence for the media’s role in obesity and unhealthy consumption is overwhelming. Not only are unhealthy foods presented more frequently, more prominently, and more positively as compared to healthy foods [15,54], experimental [19,24,28,48] and survey research [43,44] also suggests that the presentation of unhealthy foods can significantly shape unhealthy consumption habits among children.

This study demonstrates 3 things. First, the findings of this study suggest a more nuanced picture. Linking panel survey data to content analytical data on the content that children were exposed to showed that unhealthy or healthy presentation alone were not related to healthy or unhealthy consumption behaviors. Such relationships may be observable in forced-exposure experimental studies with strong stimuli and measures briefly after stimulus exposure, but not in a longitudinal study sampling actual content [55].

Second, and related to that, this study shows that the way in which foods are presented matters. Centrally placed unhealthy foods do in fact show a positive relationship to unhealthy eating behaviors; centrally placed healthy foods, however, appear to have the opposite effect. Also, healthy foods placed noncentrally seem to prime unhealthy eating habits, not healthy ones.

Third, by and large, children’s movies and TV series seem to be more likely to be positively related to unhealthy than healthy eating behaviors. Healthy consumption behaviors were completely unrelated to exposure to media content. Also, when centrality was low, even healthy foods in the media seemed to foster unhealthy consumption.

Overall, these findings inform and qualify the debate about the media’s impact on healthy and unhealthy consumption behaviors among children. Most importantly, the call to simply place more healthy foods in children’s media may, according to our findings, not be sufficient to combat unhealthy eating and childhood obesity.

## Abbreviations

REFCAM: Reactivity to Embedded Food Cues In Advertising Model

## References

[1] Jordan AB (2007). Heavy television viewing and childhood obesity. J Child Media.

[2] Boyland EJ, Whalen R (2015). Food advertising to children and its effects on diet: review of recent prevalence and impact data. Pediatr Diabetes.

[3] Russell SJ, Croker H, Viner RM (2019). The effect of screen advertising on children's dietary intake: a systematic review and meta-analysis. Obes Rev.

[4] Sadeghirad B, Duhaney T, Motaghipisheh S, Campbell NRC, Johnston BC (2016). Influence of unhealthy food and beverage marketing on children's dietary intake and preference: a systematic review and meta-analysis of randomized trials. Obes Rev.

[5] Smith R, Kelly B, Yeatman H, Boyland E (2019). Food marketing influences children's attitudes, preferences and consumption: a systematic critical review. Nutrients.

[6] Dixon HG, Scully ML, Wakefield MA, White VM, Crawford DA (2007). The effects of television advertisements for junk food versus nutritious food on children's food attitudes and preferences. Soc Sci Med.

[7] Halford JCG, Gillespie J, Brown V, Pontin EE, Dovey TM (2004). Effect of television advertisements for foods on food consumption in children. Appetite.

[8] Naderer B, Matthes J, Marquart F, Mayrhofer M (2018). Children’s attitudinal and behavioral reactions to product placements: investigating the role of placement frequency, placement integration, and parental mediation. Int J Advert.

[9] Coates AE, Hardman CA, Halford JCG, Christiansen P, Boyland EJ (2019). Social media influencer marketing and children's food intake: a randomized trial. Pediatrics.

[10] Norman J, Kelly B, McMahon A-T (2018). Sustained impact of energy-dense TV and online food advertising on children's dietary intake: a within-subject, randomised, crossover, counter-balanced trial. Int J Behav Nutr Phys Act.

[11] Vassallo AJ, Kelly B, Zhang L, Wang Z, Young S, Freeman B (2018). Junk food marketing on Instagram: content analysis. JMIR Public Health Surveill.

[12] Coates AE, Hardman CA, Halford JCG, Christiansen P, Boyland EJ (2019). Food and beverage cues featured in YouTube videos of social media influencers popular with children: an exploratory study. Front Psychol.

[13] Hurwitz LB, Morales ED, Montague H, Lauricella AR, Wartella E (2016). Mobile marketing to children: a content analysis of food and beverage company apps. Public Health.

[14] Keller SK, Schulz PJ (2011). Distorted food pyramid in kids programmes: a content analysis of television advertising watched in Switzerland. Eur J Public Health.

[15] Matthes J, Naderer B (2019). Sugary, fatty, and prominent: food and beverage appearances in children's movies from 1991 to 2015. Pediatr Obes.

[16] Naderer B, Matthes J, Spielvogel I (2019). How brands appear in children’s movies. A systematic content analysis of the past 25 years. Int J Advert.

[17] Vilaro MJ, Barnett TE, Watson AM, Merten JW, Mathews AE (2017). Weekday and weekend food advertising varies on children's television in the USA but persuasive techniques and unhealthy items still dominate. Public Health.

[18] Olafsdottir S, Berg C (2016). Food appearances in children’s television programmes in Sweden: food in children’s TV. Int J Consumer Studies.

[19] Naderer B, Matthes J, Binder A (2018). Shaping children's healthy eating habits with food placements? Food placements of high and low nutritional value in cartoons, children's BMI, food-related parental mediation strategies, and food choice. Appetite.

[20] John DR (1999). Consumer socialization of children: a retrospective look at twenty-five years of research. J Consum Res.

[21] Folkvord F, Anschütz DJ, Boyland E, Kelly B, Buijzen M (2016). Food advertising and eating behavior in children. Curr Opin Behav Sci.

[22] Alonso-Alonso M, Woods SC, Pelchat M (2015). Food reward system: current perspectives and future research needs. Nutr Rev.

[23] Binder A, Naderer B, Matthes J (2020). The effects of gain- and loss-framed nutritional messages on children's healthy eating behaviour. Public Health Nutr.

[24] Auty S, Lewis C (2004). Exploring children’s choice: the reminder effect of product placement. Psychol Mark.

[25] Matthes J, Naderer B (2015). Children’s consumption behavior in response to food product placements in movies. J Consumer Behav.

[26] Uribe R, Fuentes-García A (2015). The effects of TV unhealthy food brand placement on children. Its separate and joint effect with advertising. Appetite.

[27] Anschutz DJ, Engels R, Van Strien T (2009). Side effects of television food commercials on concurrent nonadvertised sweet snack food intakes in young children. Am J Clin Nutr.

[28] Harris JL, Bargh JA, Brownell KD (2009). Priming effects of television food advertising on eating behavior. Health Psychol.

[29] Dias M, Agante L (2011). Can advergames boost children's healthier eating habits? A comparison between healthy and non-healthy food. J Consumer Behav.

[30] Charry KM (2014). Product placement and the promotion of healthy food to pre-adolescents: when popular TV series make carrots look cool. Int J Adv.

[31] Folkvord F, de Bruijne M (2020). The effect of the promotion of vegetables by a social influencer on adolescents' subsequent vegetable intake: a pilot study. Int J Environ Res Public Health.

[32] Binder A, Naderer B, Matthes J (2021). Shaping healthy eating habits in children with persuasive strategies: toward a typology. Front Public Health.

[33] Folkvord F (2019). The Psychology of Food Marketing and (Over)eating.

[34] Kamleitner B, Khair Jyote A (2013). How using versus showing interaction between characters and products boosts product placement effectiveness. Int J Advert.

[35] Naderer B, Matthes J, Zeller P (2018). Placing snacks in children’s movies: cognitive, evaluative, and conative effects of product placements with character product interaction. Int J Advert.

[36] Li C, Ademiluyi A, Ge Y, Park A (2022). Using social media to understand web-based social factors concerning obesity: systematic review. JMIR Public Health Surveill.

[37] Birch LL, Fisher JO (1998). Development of eating behaviors among children and adolescents. Pediatrics.

[38] Sasson H, Mesch GS, Hobbs R, Mihailidis P (2019). The International Encyclopedia of Media Literacy.

[39] Nathanson AI (2001). Mediation of children’s television viewing: working toward conceptual clarity and common understanding. Ann Int Commun Assoc.

[40] Warren R, Hobbs R, Mihailidis P (2019). The International Encyclopedia of Media Literacy.

[41] Valkenburg PM, Krcmar M, Peeters AL, Marseille NM (1999). Developing a scale to assess three styles of television mediation: “instructive mediation,” “restrictive mediation,” and “social Coviewing”. Journal of Broadcasting & Electronic Media.

[42] Cox R, Skouteris H, Rutherford L, Fuller-Tyszkiewicz M, Dell’Aquila D, Hardy LL (2012). Television viewing, television content, food intake, physical activity and body mass index: a cross-sectional study of preschool children aged 2–6 years. Health Promot J Aust.

[43] Emond JA, Longacre MR, Drake KM (2019). Influence of child-targeted fast food TV advertising exposure on fast food intake: a longitudinal study of preschool-age children. Appetite.

[44] Fuller-Tyszkiewicz M, Skouteris H, Hardy LL, Halse C (2012). The associations between TV viewing, food intake, and BMI. A prospective analysis of data from the Longitudinal Study of Australian Children. Appetite.

[45] (2016). Obesity and overweight. World Health Organization.

[46] De Decker A, Sioen I, Verbeken S, Braet C, Michels N, De Henauw S (2016). Associations of reward sensitivity with food consumption, activity pattern, and BMI in children. Appetite.

[47] Hayes AF, Matthes J (2009). Computational procedures for probing interactions in OLS and logistic regression: SPSS and SAS implementations. Behav Res Methods.

[48] Folkvord F, Anschütz DJ, Buijzen M, Valkenburg PM (2013). The effect of playing advergames that promote energy-dense snacks or fruit on actual food intake among children. Am J Clin Nutr.

[49] Maller O, Turner RE (1973). Taste in acceptance of sugars by human infants. J Comp Physiol Psychol.

[50] Harris G, Thomas A, Booth DA (1990). Development of salt taste in infancy. Dev Psychol.

[51] Binder A, Naderer B, Matthes J (2019). Do children’s food choices go with the crowd? Effects of majority and minority peer cues shown within an audiovisual cartoon on children’s healthy food choice. Soc Sci Med.

[52] Hang H, Davies I, Schüring J (2020). Children's conformity to social norms to eat healthy: a developmental perspective. Soc Sci Med.

[53] Matthes J, Naderer B (2016). Product placement disclosures: exploring the moderating effect of placement frequency on brand responses via persuasion knowledge. Int J Advert.

[54] Araque-Padilla R, Villegas-Navas V, Montero-Simo MJ (2019). Non-branded food placements in children's entertainment programs: a content analysis. Health Commun.

[55] Arendt F, Naderer B, Abdollahi M, Mittelberger A, Surzhyk O, Zhou L (2015). Television commercials and fading behavioral brand choice effects in Austrian children. J Child Media.

